# The clinical impacts of postoperative complications after colon cancer surgery for the clinical course of adjuvant treatment and survival

**DOI:** 10.1007/s10147-023-02332-y

**Published:** 2023-04-11

**Authors:** Toru Aoyama, Koji Oba, Michitaka Honda, Masaru Muto, Shuhei Mayanagi, Hiromichi Maeda, Mitsuro Kanda, Kosuke Kashiwabara, Junichi Sakamoto, Takaki Yoshikawa

**Affiliations:** 1 Department of Surgery, Yokohama City University, 3-9 Fukuura, Kanazawa-ku, Yokohama, 236-0004 Japan; 2 Department of Biostatistics, The University of Tokyo, Tokyo, Japan; 3 Department of Minimally Invasive Surgical and Medical Oncology, Fukushima Medical University, Fukushima, Japan; 4 Japanese Foundation for Multidisciplinary Treatment of Cancer, Tokyo, Japan; 5 Division of Esophageal Surgery, Shizuoka Cancer Center Hospital, Shizuoka, Japan; 6 Department of Surgery, Kochi University, Kochi, Japan; 7 Department of Gastroenterological Surgery, Nagoya University Graduate School of Medicine, Nagoya, Japan; 8 Tokai Central Hospital, Gifu, Japan; 9 Department of Gastric Surgery, National Cancer Center Hospital, Tokyo, Japan; 10 Data Science Office, Clinical Research Promotion Center, The University of Tokyo Hospital, Tokyo, Japan

**Keywords:** Colon cancer, Surgical complication, Adjuvant treatment, Survival

## Abstract

**Aim:**

We investigated whether or not postoperative complications (POCs) themselves have a negative survival impact or indirectly worsen the survival due to insufficient adjuvant chemotherapy in a pooled analysis of two large phase III studies performed in Japan

**Patients and methods:**

The study examined the patients who enrolled in 1304, phase III study comparing the efficacy of 6 and 12 months of capecitabine as adjuvant chemotherapy for stage III colon cancer patients and in 882, a phase III study to confirm the tolerability of oxaliplatin, fluorouracil, and l-leucovorin in Japanese stage II/III colon cancer patients. In our study, POCs were defined as the following major surgical complications: anastomotic leakage, pneumonia, bowel obstruction/ileus, surgical site infection, postoperative bleeding, urinary tract infection, and fistula. Patients were classified as those with POCs (C group) and those without POCs (NC group).

**Results:**

A total of 2095 patients were examined in the present study. POCs were observed in 169 patients (8.1%). The overall survival (OS) rates at 5 years after surgery were 75.3% in the C group and 86.5% in the NC group (*p* = 0.0017). The hazard ratio of POCs for the OS in multivariate analysis was 1.70 (95% confidence interval, 1.19 to 2.45; *p* = 0.0040). The time to adjuvant treatment failure (TTF) of adjuvant chemotherapy was similar between the groups, being 68.6% in the C group and 67.1% in the NC group for the 6-month continuation rate of adjuvant chemotherapy. The dose reduction rate of adjuvant chemotherapy and adjuvant treatment suspension rate were also similar between the groups (C vs. NC groups: 45.0% vs. 48.7%, *p* = 0.3520; and 52.7% vs. 55.0%, *p* = 0.5522, respectively).

**Conclusion:**

POCs were associated with a poor prognosis but did not affect the intensity of adjuvant chemotherapy. These results suggested that POCs themselves negatively influence the survival.

## Introduction

Colorectal cancer is the third-most common cancer and the second leading cause of cancer-related death in the world [1, 2]. Curative resection followed by postoperative adjuvant chemotherapy is a standard treatment for locally advanced colon cancer [3, 4, 5]. Although the survival rate achieved by surgery and adjuvant treatment is gradually increasing, about 30–40% of patients experience recurrence, even after this multimodality treatment [6, 7].

Recently, postoperative surgical complications (POCs) for colon cancer have been reported to affect patients’ survival [8, 9, 10]. Previous studies have shown that the presence of POCs is an independent risk factor associated with a worse overall survival (OS) and an increased overall recurrence rate [8]. In previous studies, cytokines released by inflammation in POCs, such as anastomotic leakage, abdominal abscess, or pneumonia, were suggested to potentially play a significant role in tumor progression or metastasis, which would worsen the prognosis [11, 12]. However, most previous studies only examined the surgery data and the survival, ignoring the effects of adjuvant chemotherapy. It is likely that patients who develop POCs cannot initiate or are late to start adjuvant chemotherapy, as recovery from POCs can take months. Indeed, two reports clarified that the negative impact of POCs was associated with the omission of or a delay in adjuvant chemotherapy [13, 14].

To clarify whether or not POCs themselves have a negative survival impact or indirectly worsen the survival due to insufficient adjuvant chemotherapy, we examined the impact of POCs on the survival and the intensity of adjuvant chemotherapy in patients who initiated adjuvant chemotherapy as scheduled in phase III studies of adjuvant chemotherapy.

## Patients and methods

### Patients

Patients’ data and oncological outcomes registered in two clinical trials of Japanese Foundation of Multidisciplinary Treatment of Cancer (JFMC) studies (37 and 41) were pooled [15, 16].

### JFMC trials in the pooled analysis

The JFMC 37 trial was a multi-institutional randomized control phase III trials to compare the efficacy of 6 and 12 months of capecitabine as adjuvant chemotherapy for stage III colon cancer patients. In the JFMC 37 trial, 1304 patients were registered from 333 Japanese institutions. Among them, 654 patients were registered to the 6-month adjuvant chemotherapy group, and 650 were registered to the 12-month adjuvant chemotherapy group. Details of adjuvant treatment were described in a previous report [15].

The JFMC-41 trial was a non-randomized open label trial to confirm the tolerability of oxaliplatin, fluorouracil, and L-leucovorin in Japanese stage II/III colon cancer patients. A total of 882 patients were registered in this trial. Details of adjuvant treatment were described in a previous report [16].

### Definition of POCs

Each study pre-specified that physicians must report any surgical morbidity at study enrollment. All information on complications was extracted from the case-report forms for each trial. In the present study, POCs were defined as the following major surgical complications: anastomotic leakage, pneumonia, bowel obstruction/ileus, surgical site infection, postoperative bleeding, urinary tract infection, and fistula. Each surgical complication was evaluated by each surgeon of each institution. The patients were classified based on the presence of POCs (C group) or their absence (NC group).

### Evaluations and statistical analyses

The background characteristics of postoperative clinical and pathological parameters between the C group and NC group were compared using the Mann–Whitney test or χ^2^ test. Overall survival (OS) was defined as the period between the surgery and any cause of death. The relapse-free survival (RFS) was defined as the period between the surgery and recurrence, or death, whichever came first. The data for patients who had not experienced an event were censored as of the date of the final observation.

Time-to-adjuvant treatment failure (TTF) is the interval from adjuvant chemotherapy initiation to premature discontinuation within 6 months after enrollment in the study. Treatment failure was defined as chemotherapy discontinuation prior to completion of the planned cycles for any reason, including cancer progression, adverse events, or patient choice. The OS curves, RFS curves, and TTF curves were calculated using the Kaplan–Meier method and were compared by the log-rank test. A Cox proportional hazards model was used to perform the univariable and multivariable analyses for the OS and RFS. Nine possible confounders between POCs and outcomes (age, gender, performance status, T stage, lymph node metastases, histology, lymph node dissection and treatment group) were investigated in univariate analysis and significant factors were analyzed in the multivariable Cox proportional hazards model built using stepwise selection with an inclusion criterion of *P* = 0.2 to enter the model and an exclusion criterion of *P* = 0.05 to exit. A two-sided *P* < 0.05 was defined as being statistically significant.

The SAS version 9.4 (SAS Institute Inc., Cary, NC, USA) was used for all statistical analyses. This study was approved by the ethics committee of the JFMC.

## Results

### Patients’ background characteristics

**Fig. 1:**
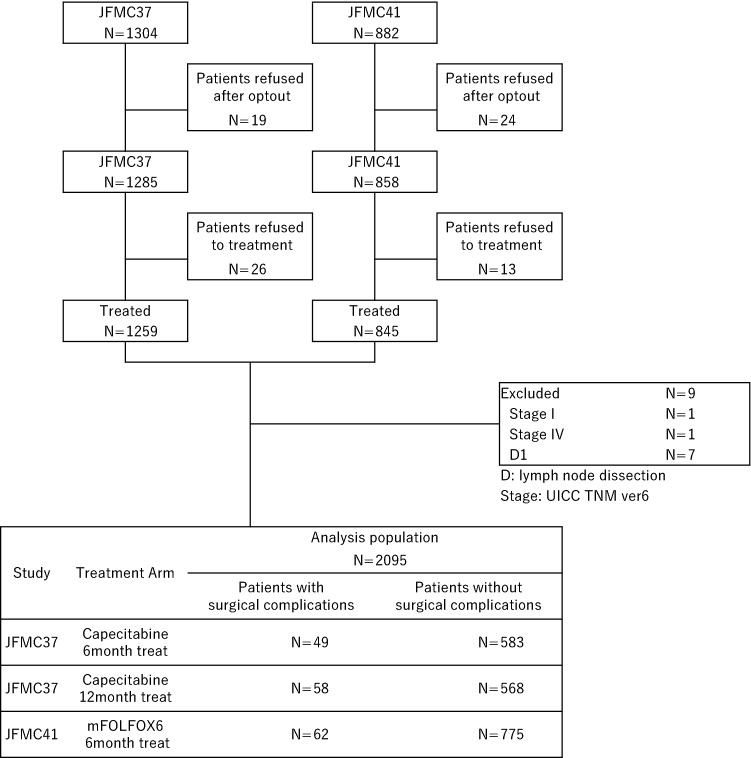
Consort diagram of the present study

A total of 2186 patients were registered to both clinical trials. Among them, we evaluated 2095 patients in the present study. The consort diagram is shown in Fig. 1. POCs were observed in 169 patients (8.1%). Bowel obstruction and surgical site infection were the most frequent POCs (2.3% and 2.1%, respectively) (Table 1). When comparing the patient’s background between C and NC groups, significant differences were observed in gender, age, and T stage (Table 2). The proportion of men, elderly subjects, and cases of advanced T stage were significantly higher in the C group than in the NC group. Furthermore, the period from surgery to the initiation of adjuvant chemotherapy was significantly longer in the C group than in the NC group (*p* < 0.0001), but the difference of median was small (39 vs 35 days).

**Table 1:** Details of postoperative complications

	Number of the patients	(%)
Bowel obstruction/Ileus	48	2.3
Surgical site infection (SSI)	45	2.1
Anastomotic leakage	27	1.3
Abdominal abscess	8	0.4
Postoperative bleeding	4	0.2
Pneumonia	4	0.2
Liver function damage	3	0.1
Renal function damage	3	0.1
Others	27	1.3

**Table 2:** Comparison clinicopathological factors between the patients with surgical complications group (C group) and the patients without surgical complications group (NC group)

Factors	All cases (*n* = 2095)		C group (*n* = 169)		NC group (*n* = 1926)		*P* value
	Number	(%)	Number	(%)	Number	(%)	
Gender							0.0234
Male	1127	53.8	105	62.1	1022	53.1	
Female	968	46.2	64	37.9	904	46.9	
Age (years)							0.0248
< 70	1461	69.7	105	62.1	1356	70.4	
≥ 70	634	30.3	64	37.9	570	29.6	
PS (ECOG)							0.2854
0	1994	95.2	158	93.5	1836	95.3	
1	101	4.8	11	6.5	90	4.7	
Histology							0.2475
Well-mod	1937	92.5	152	89.9	1785	92.7	
Poor	154	7.4	16	9.5	138	7.2	
Not classified	4	0.2	1	0.6	3	0.2	
UICC T stage							0.0379
T1–T3	1473	70.3	107	63.3	1366	70.9	
T4	622	29.7	62	36.7	560	29.1	
LN node metastases							0.1492
N0–1	1551	74.0	133	78.7	1418	73.6	
N2	544	26.0	36	21.3	508	26.4	
Stage UICC 6th							0.0871
Stage IIA	97	4.6	8	4.7	89	4.6	
Stage IIB	56	2.7	6	3.6	50	2.6	
Stage IIIA	236	11.3	11	6.5	225	11.7	
Stage IIIB	1162	55.5	108	63.9	1054	54.7	
Stage IIIC	544	26.0	36	21.3	508	26.4	
Lymph node dissection							0.8155
D2	411	19.6	32	18.9	379	19.7	
D3	1684	80.4	137	81.1	1547	80.3	
Treatment Group							0.4093
JFMC37 6-month	632	30.2	49	29.0	583	30.3	
JFMC37 12-month	626	29.9	58	34.3	568	29.5	
JFMC-41	837	40.0	62	36.7	775	40.2	

*ECOG* Eastern Cooperative Oncology Group, *Well-Mod*: well-moderately differentiated, *Por* poorly differentiated, *UICC* Union for International Cancer Control, *LN* lymph node

### A survival analysis between the C and NC groups

**Fig. 2:**
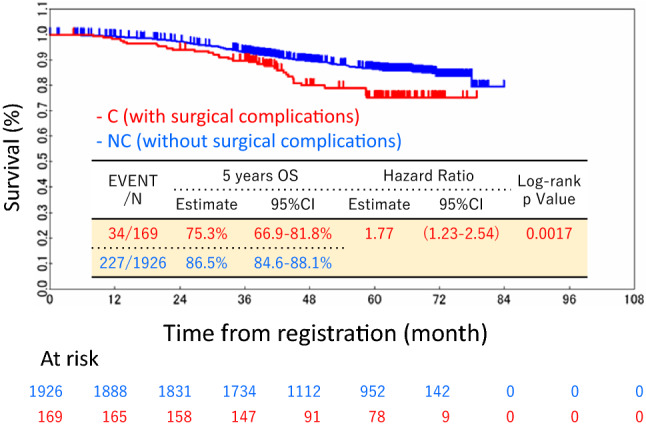
The overall survival curves of the C group (those with surgical complications) and the NC group (those without surgical complications)

**Fig. 3:**
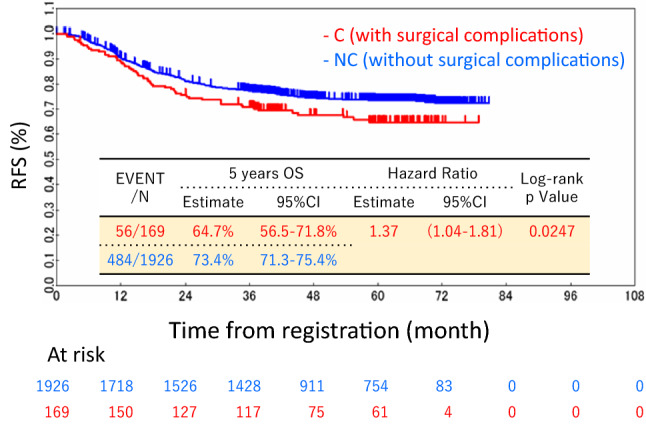
The disease-free survival curves of the C group (those with surgical complications) and the NC group (those without surgical complications)

The crude OS rates at 5 years after surgery were 75.3% in the C group and 86.5% in the NC group (*p* = 0.0017). The Kaplan–Meier curves of OS are shown in Fig. 2. Both crude and multivariable analyses showed that POCs were a significant risk factor associated with a poor OS (Table 3). The adjusted hazard ratio for the OS was 1.70 (95% confidence interval, 1.19–2.45; *p* = 0.0040). The crude RFS rates at 5 years after surgery were 64.7% in the C group and 73.4% in the NC group (*p* = 0.0247). The Kaplan–Meier curves of RFS are shown in Fig. 3. Similar to the OS, both univariable and multivariable analyses showed that POCs were significant factors associated with a poor RFS. The adjusted hazard ratio for the RFS was 1.36 (95% confidence interval, 1.03–1.79; *p* = 0.0309).

**Table 3:** Uni and Multivariate Cox proportional hazards analysis of clinicopathological factors for overall survival

Factors	Number	Univariate analysis			Multivariate analysis		
		HR	95%CI	*P* value	HR	95%CI	*P* value
Age (years)				0.0022			0.0035
– < 70	1461	1.00			1.00		
70≦–	634	1.47	1.15–1.89		1.45	1.13–1.86	
Gender				0.3022			
Male	1127	1.00					
Female	968	0.88	0.69–1.12				
PS (ECOG)				0.9931			
0	1994	1.00					
1	101	1.00	0.56–1.78				
UICC T status				< 0.0001			< 0.0001
T1–T3	1473	1.00			1.00		
T4	622	2.42	1.90–3.08		2.11	1.65–2.70	
Lymph node metastases				< 0.0001			< 0.0001
N0–1	1551	1.00			1.00		
N2	544	2.40	1.87–3.07		2.17	1.69–2.80	
Histology				< 0.0001			0.0014
Well-moderate	1937	1.00			1.00		
Poor	154	2.22	1.55–3.19		1.81	1.26–2.60	
Surgical complication				0.0017			0.0040
No	1926	1.00			1.00		
Yes	169	1.77	1.23–2.54		1.70	1.19–2.45	
Lymph node dissection				0.2741			
D2	411	1.00					
D3	1684	1.19	0.87–1.64				
Treatment arm				0.1139			
JFMC37 6-month	632	1.00					
JFMC37 12-month	626	0.75	0.56–1.00				
JFMC-41	837	0.99	0.72–1.35				

*ECOG* Eastern Cooperative Oncology Group, *UICC* Union for International Cancer Control, *Multivariate analysis* Selected by stepwise method from significant factors by univariate analysis

### Intensity of adjuvant treatment between the C and NC groups

**Fig. 4:**
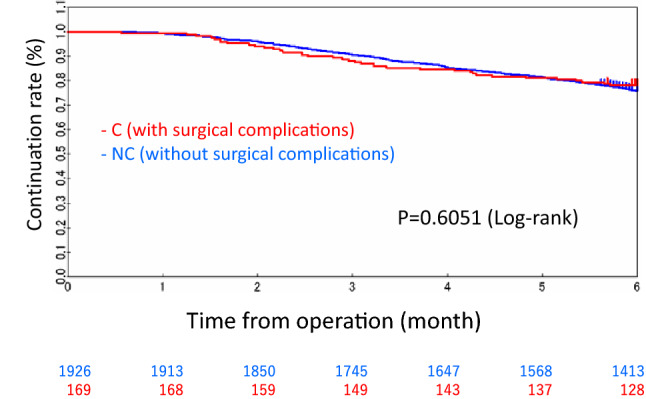
The continuation of adjuvant treatment between the C group (those with surgical complications) and the NC group (those without surgical complications)

The TTF at 6 months in the C group and the NC group (Fig. 4), showing no significant differences between the groups (*p* = 0.6051). The 6-month continuation rate of adjuvant chemotherapy was 68.6% in C group and 67.1% in NC group. The dose reduction rate of adjuvant chemotherapy and adjuvant treatment suspension rate were also similar between the groups (C vs. NC: 45.0% vs. 48.7%, *p* = 0.3520; and 52.7% vs. 55.0%, *p* = 0.5522, respectively) (Table 4). In addition, similar results were observed when separating the JFMC 37 and JFMC-41 trial. In 6-month treatment group of JFMC 37 trial, continuation rate of adjuvant chemotherapy was 75.5% in C group and 71.4% in NC group. There were not significantly differences between two groups (*p* = 0.5352). Dose reduction rate of adjuvant chemotherapy and adjuvant treatment suspension rate were similar between C group and NC group (38.8% vs 37.0%, *p* = 0.8103 and 34.7% vs 32.2%, *p* = 0.7253, respectively). In 12-month treatment group of JFMC 37 trial, continuation rate of adjuvant chemotherapy was 41.4% in C group and 46.8% in NC group. There were not significantly differences between two groups (*p* = 0.4277). Dose reduction rate of adjuvant chemotherapy and adjuvant treatment suspension rate were similar between C group and NC group (39.7% vs 48.4%, *p* = 0.2032 and 43.1% vs 45.1%, *p* = 0.7742, respectively). In JFMC-41, 6-month continuation rate of adjuvant chemotherapy was 69.4% in C group and 64.9% in NC group. There were not significantly differences between two groups (*p* = 0.4788). Dose reduction rate of adjuvant chemotherapy and adjuvant treatment suspension rate were similar between C group and NC group (59.7% vs 63.5%, *p* = 0.5498 and 77.4% vs 84.1%, *p* = 0.1694).

**Table 4:** Comparison postoperative adjuvant treatment course between the patients with surgical complications group (C group) and the patients without surgical complications group (NC group)

Factors	All cases (*n* = 2095)		C group (*n* = 169)		NC group (*n* = 1926)		*P* value
	Number	(%)	Number	(%)	Number	(%)	
Discontinuation							0.6893
No	1409	67.3	116	68.6	1293	67.1	
Yes	686	32.7	53	31.4	633	32.9	
Dose reduction							0.3520
No	1081	51.6	93	55.0	988	51.3	
Yes	1014	48.4	76	45.0	938	48.7	
Dose suspension							0.5522
No	946	45.2	80	47.3	866	45.0	
Yes	1149	54.8	89	52.7	1060	55.0	

## Discussion

The present study explored whether or not postoperative complications (POCs) themselves have a negative survival impact or indirectly worsen the survival due to insufficient adjuvant chemotherapy in patients who underwent curative resection for colon cancer. The major finding of the present study was that POCs were associated with a poor prognosis but did not affect the intensity of adjuvant chemotherapy. These results strongly suggest that the POCs themselves negatively influence the survival.

First, we wish to discuss the clinical influence of POCs on the long-term oncological outcomes. Initially, we thought that the low frequency of postoperative surgical complications and the lack of association between postoperative surgical complications and adjuvant treatment made it unlikely that surgical complications would affect prognosis. However, there were significantly differences in long-term oncological outcomes between C group and NC group. In addition, the hazard ratio of postoperative surgical complications was similar to previous studies. Moreover, previous study demonstrated that even grade I postoperative surgical complications according to C-D classification had clinical effects for poor oncological outcomes in colorectal cancer [17, 18]. Thus, regardless of the severity of the postoperative surgical complication, the occurrence of the postoperative complication itself may have affected the patient’s prognosis. Therefore, it is necessary to minimize the postoperative complications after surgery. These are added in the discussion section. So, what should physicians consider based on the present results? First proposal is to give more intensive chemotherapy for the patients who developed surgical complications but recovered soon and were ready to start adjuvant chemotherapy. Physicians should treat these patients as the patients with high recurrent risk. The current Japanese colorectal cancer guideline described that physicians should select observation, fluoropyrimidine monotherapy, or oxaliplatin-based doublet regimen for stage II and mono or doublet regimen for stage III, considering the recurrent risk. Second proposal is to do not hesitate adjuvant chemotherapy for these patients. Physicians may hesitate adjuvant chemotherapy for these patients by being afraid of poor compliance. However, such afraid is meaningless because compliance of chemotherapy is not disturbed in these patients as shown in this study. Thus, special management is unnecessary for these patients. On the other hands, other possibilities of the worse prognosis in the C group were differences of patient’s background. There were older men, worse performance status, and more advanced cancer in the C group. These differences might also affect for the long-term oncological outcomes.

Next, we examined intensity of adjuvant chemotherapy. Unexpectedly, POCs did not affect the TTF, continuation rate, dose reduction, or suspension of adjuvant chemotherapy. These results suggested that the poor prognosis found in cases with POCs was not due to the intensity of adjuvant chemotherapy. Tevis et al. retrospectively identified the risk factors associated with delays in chemotherapy after rectal cancer surgery and evaluated the effects of delayed therapy on long-term outcomes [19]. They found that postoperative complications and 30-day readmissions were associated with delays in chemotherapy ≥ 8 weeks after surgery. Patients who received chemotherapy ≥ 8 weeks postoperatively were found to have worse local and distant recurrence rates and a worse OS than patients who received chemotherapy within 8 weeks of surgery. Furthermore, a recent meta-analysis of adjuvant therapy treatment times in colorectal cancer revealed that a 4-week treatment delay was associated with reductions in both the OS and DFS [20]. However, the period from the surgery to the initiation of adjuvant chemotherapy in the C group was similar to that in the NC group (39 vs. 35 days, median). In addition, Merkow et al. evaluated the association of postoperative complications with the administration of adjuvant therapy after colectomy for cancer [13]. In their study, the strongest predictors of adjuvant chemotherapy omission were prolonged postoperative ventilation, renal failure, reintubation, and pneumonia. Superficial surgical site infection did not decrease adjuvant therapy receipt but did delay the time to adjuvant treatment use by three-fold. Severe complications increased time to chemotherapy by 65%. Abscess or anastomotic leak increased time to adjuvant chemotherapy by more than five-fold. However, these differences were not observed in the present study. The POCs in our study may have been milder than those in previous studies. Therefore, these POCs did not affect the clinical course of adjuvant chemotherapy.

Regarding whether or not POCs markedly influence the survival, Beck et al. evaluated the clinical impact of POCs on the long-term oncological outcomes in 2158 colorectal cancer patients with regard to their severity according to the Clavien-Dindo classification (CDC) [21]. In their study, 467 patients (21.6%) had POCs, including grade I in 141 (6.5%), grade II in 162 (7.5%), grade III in 112 (5.2%), and grade IV in 52 (2.4%). They found that even patients with grade I POCs had a significantly worse prognosis than those without any surgical complications. Thus, the prognostic impact of POCs is evident, regardless of the severity.

In the present cohort, the incidence of the surgical complications was 8.5% in the present study, while 15–25% in the previous pivotal studies [22]. These discrepancies might be due to the differences of the clinical trial. The present study focused on the efficacy of the postoperative adjuvant chemotherapy. Thus, the physicians could not enroll the patients who developed postoperative surgical complications and did not recover until initiating adjuvant chemotherapy defined by the trial.

Several limitations associated with the present study warrant mention. First, the severity of the POCs was unclear. Previous studies showed that the occurrence of severe POCs was a risk factor for delayed adjuvant treatment. However, the duration between the day of surgery and the start of adjuvant chemotherapy was similar between the NC and C groups in the present study. Thus, the severity of the POCs in our study was not very high. Second, in the present study, we evaluated the clinical relationship between POCs and cytotoxic agents. Recently, molecular-targeted agents and immune checkpoint inhibitors have been introduced as adjuvant treatment in colorectal cancer [23, 24]. However, the present study did not focus on this issue.

In conclusion, POCs were associated with a poor prognosis but did not affect the intensity of adjuvant chemotherapy. These results suggest that the POCs themselves have a negative survival impact.

## Data Availability

The data that support the findings of this study are available from the corresponding author upon reasonable request.
